# Experimental Study on LTE Mobile Network Performance Parameters for Controlled Drone Flights

**DOI:** 10.3390/s24206615

**Published:** 2024-10-14

**Authors:** Janis Braunfelds, Gints Jakovels, Ints Murans, Anna Litvinenko, Ugis Senkans, Rudolfs Rumba, Andis Onzuls, Guntis Valters, Elina Lidere, Evija Plone

**Affiliations:** 1 Institute of Photonics, Electronics and Telecommunications, Riga Technical University, LV-1048 Riga, Latvia; ints.murans@rtu.lv (I.M.); anna.litvinenko@rtu.lv (A.L.); ugis.senkans@rtu.lv (U.S.); 2 Ventures Department, Latvijas Mobilais Telefons SIA, LV-1026 Riga, Latvia; gints.jakovels@lmt.lv (G.J.); guntis.valters@lmt.lv (G.V.); evija.plone@lmt.lv (E.P.); 3 Institute of Applied Computer Systems, Riga Technical University, LV-1048 Riga, Latvia; rudolfs.rumba@rtu.lv; 4 Technical Department, Latvijas Mobilais Telefons SIA, LV-1026 Riga, Latvia; andis.onzuls@lmt.lv; 5 Institute of Information Technology, Riga Technical University, LV-1026 Riga, Latvia; elina.lidere@lmt.lv; 6 Marketing Communication Department, Latvijas Mobilais Telefons SIA, LV-1026 Riga, Latvia

**Keywords:** unmanned aerial vehicle, drone, mobile network, LTE, reference signal received power, reference signal received quality, signal to interference and noise ratio, uplink, downlink, ping

## Abstract

This paper analyzes the quantitative quality parameters of a mobile communication network in a controlled drone logistic use-case scenario. Based on the analysis of standards and recommendations, the values of key performance indicators (KPIs) are set. As the main network-impacting parameters, reference signal received power (RSRP), reference signal received quality (RSRQ), and signal to interference and noise ratio (SINR) were selected. Uplink (UL), downlink (DL), and ping parameters were chosen as the secondary ones, as they indicate the quality of the link depending on primary parameters. The analysis is based on experimental measurements performed using a Latvian mobile operator’s “LMT” JSC infrastructure in a real-life scenario. To evaluate the altitude impact on the selected network parameters, the measurements were performed using a drone as transport for the following altitude values: 40, 60, 90, and 110 m. Network parameter measurements were implemented in automatic mode, allowing switching between LTE4–LTE2 standards, providing the opportunity for more complex analysis. Based on the analysis made, the recommendations for the future mobile networks employed in controlled drone flights should correspond to the following KPI and their values: −100 dBm for RSRP, −16 dB for RSRQ, −5 dB for SINR, 4096 kbps for downlink, 4096 kbps for uplink, and 50 ms for ping. Lastly, recommendations for a network coverage digital twin (DT) model with integrated KPIs are also provided.

## 1. Introduction

More and more innovative and advanced technologies are being introduced into modern-day life. One such technology that has recently experienced an increase in topicality is drones, also known as unmanned aerial vehicles (UAVs). The manufacture of UAVs and their integration within the new industrial sphere show an increase in potential and business aspects with current forecasts indicating a market share of around USD 100 billion in years to come [1]. The realization potential of UAVs is vast as new applications are constantly being invented and presented. There are multiple areas in which UAVs have especially been used lately. Some of those are monitoring [2,3,4,5], inspections [6,7], examinations [8,9], mapping [10,11], agriculture [10,12], surveillance [13,14], logistics [15,16], the transport sector [8,14,17], military industry [18,19], communications and newsgathering [11,20] and media, as well as solutions for research needs [20,21]. Also, another type of application that may encounter a high number of requests for UAVs in the upcoming years is package delivery [22,23]. Within the U.S., multiple companies have recently been investigating the package delivery capabilities of UAVs [11].

Overall, in the civilian and governmental sector, one of the crucial use cases is disaster response and recovery [24,25,26], thus ensuring a rapid reaction time by the response teams, so that rescuers and special services utilize real-time information observed prior to their arrival [27]. Typically, the operational scheme of how UAVs are used is by realizing a line-of-sight (LOS) local type of connection between the UAV and its operator—the end-user. Therefore, the communication link with the UAV is also local, typically Wi-Fi-based technology, and tends to operate by using unlicensed frequency bands. This application limits the operation of those UAVs to regions where the UAV can be used locally [28,29], without realizing necessary coordination with the responsible institution that ensures the necessary permits. However, when a licensed frequency spectrum is used, research conducted [30] regarding the available radio link technologies that are utilized in UAV operations indicated three alternatives or choices for UAV datalink coverage. Such techniques include terrestrial mobile 4G/long-term evolution (LTE) networks, satellite links within the allocated spectrum, or 5G networks (if available and applicable) that are adapted to air–ground usage, or UAV terrestrial networks using licensed spectrum in very high frequency (VHF) or ultra-high frequency (UHF) bands in specific situations.

Furthermore, the realization of UAVs in civilian and commercial fields has shown essential topicality in recent years. However, for advanced applications of UAVs, an extended and increased, beyond visual-line-of-sight, configuration is required. In comparison to the typical LOS realizations, such extended services require fulfillment of the regulatory standards; thus, a radio communication link has to be established correctly and should maintain stable and reliable link connections. As a means of transmission link media, long-term evolution UMTS (LTE) is a core standard in cellular systems for the radio communication links of UAVs [31].

Multiple civil applications for drones in different industries have led to an increasing demand for a stable and suitable level of performance in wireless connectivity to UAVs. Hence, mobile networks ensure a relatively reliable base for secure wide-area connectivity to such devices. However, there still is a requirement for ubiquitous connectivity for a wide range of other user equipment (UE) that utilizes the available frequency spectrum that needs to be taken into account. For instance, sensors, driverless cars, and enhanced mobile broadband (eMBB) devices. All of these require the use of airborne communication capabilities. Therefore, aerial nodes of various types can be developed and adjusted for the necessary performance improvements, flexibility, and agility of the 5G and other next-generation mobile networks [32].

Moreover, two main factors [32] have to be taken into account regarding the UAVs and their connection with the wireless networks. One of them is related to the wireless networks’ advancement to support the professional or private use of UAVs. Another one is how UAVs could support the performance of and improvement in the wireless networks themselves. For instance, to increase the capacity and availability of wireless networks when it is necessary by enlarging the operational coverage range, enhancing agility and availability using UAVs as aerial nodes.

## 2. Overview of Drone Realization for LTE Mobile Network Parameter Measurements

The overview of studies that describe certain drone usage for certain LTE mobile network parameter measurements is included in Table 1.

The topicality of our research also supports recent studies that measure and evaluate certain important LTE mobile network parameters. For instance, a [33] study in Aalborg (Denmark) showed the development of a setup where a crane was used for lifting a multi-antenna receiver. This was carried out within the city, its suburbs, rural areas as well as in the industrial part of the city. The multi-antenna receiver was raised from ground level to 40 m and measurements of the received signal power, RSRP, RSRQ, and SINR were measured. Another approach was shown in [31] to observe radio interference for UAV connectivity. In this research, a Rohde & Schwarz mobile network scanner was mounted underneath a commercial hexacopter drone to measure RSRP, DL, UL, and others within 15–120 m. In turn, the authors of the research in [34] used a portable radio network scanner that was attached underneath a commercial UAV to detect airborne UEs around the altitude of 15–120 m and observe RSRP, RSRQ, and other parameters. Other research [35] used a quad-rotor drone with an open-source autopilot platform and a 4G-only mobile phone for measuring latitudes, longitudes, heights of the drone, and the RSRP of a 4G network up to 500 m of altitude within an urban environment. The research in [28] showed public LTE network measurements with drones and smartphones in a rural environment while investigating UL parameters at 50 to 100 m of altitude.

Another interesting approach can be observed in the research of [36], where an ultra-micro drone with a 4G modem was used to measure the RSSI parameter at heights of 30–120 m to create a drone-based mapping of 4G cellular network coverage. A topical study with regard to our own is shown in [37] where a commercial drone is used with a smartphone that has a portable mobile-network-testing application installed. The research observes RSRP, RSRQ, RSSI, SINR, downlink throughput, and uplink throughput parameters within different altitudes and drone movement speeds. There is also novel research [38] that discusses the signal strength of 5G, LTE, and LTE-M coverage for drone communications. RSRP and SINR parameters are evaluated at heights of more than 120 m. Specific focus on the SINR delay measurements is made in [39] where a UAV that is controlled by a configured application is used to evaluate the delay measurements of drones connecting to commercial LTE and 5G networks. Additionally, the research in [40] shows the development of a model with a UAV that has a downward RX antenna and software-defined radio (SDR) software installed. The impact of a 3D antenna radiation pattern in UAV air-to-ground path loss is modeled, as well as RSRP parameters measured at altitudes of 30–110 m. Investigation of the applicability of LTE-M for network identification of unmanned aerial systems was shown in [41], where RSSI, RSRP, and RSRQ parameters were also measured at heights of 20–100 m. Lastly, the authors of the research in [42] described the development of a multirotor drone with a smartphone and drive-test application where RSRP and RSRQ parameter prediction models were described, and data from 65 to 125 m heights were shown.

Based on the evaluated previous research [28,31,33,34,35,36,37,38,39,40,41,42], 3GPP [43] recommendations, RTU and LMT previous experience, our experimental study, and data gathered and shown within this article, we have raised KPIs (see Section 3) for drone logistics using the LTE mobile network.

From the research covered in the field of drone logistics and their wireless 4G and 5G coverage communication links, it is clear that different measurement applications have been studied and tested; however, there are no clear KPIs set for suitable network performance and the corresponding parameters that would be classified as acceptable for drone logistics and utilization. If there are some KPIs defined, they are usually not analyzed in-depth as to why specifically such values and rates have been set. In this research, the authors tackle this issue by analyzing large amounts of data to estimate appropriate KPIs, giving a detailed evaluation.

Given the information mentioned in the previous paragraph, in order to improve the UAVs’ (or in other words—drones’) logistics as well as study their experimental performance in 4G and 5G datalink environments (and their corresponding parameters), it is important to carry out extensive real-time measurements while applying different use-case conditions. To achieve this, in this research, firstly we evaluated the appropriate industry practices, standards, and network parameters. Based on the collected and analyzed data, together with the Latvian mobile operator “LMT”, we defined the main KPI indicators for the network’s availability measurement and evaluation process. Then, experimental measurement setup, methodology, and application scenarios were developed and tested. Afterward, a vast amount of reference signal received power (RSRP), reference signal received quality (RSRQ), signal interference and noise ratio (SINR), uplink (UL), and downlink (DL) latency data was collected for the same route for four different altitude levels in the “LMT” mobile network. Measurements are carried out using a specially configured DJI M30 drone that carries the OnePlus NORD BE2029 mobile phone and Enhancell Echo One mobile network analyzer software. The gathered data and analysis made have a significant value for both mobile network infrastructure developers and mobile operators as well as the drone (UAV) manufacturing industry and logistic planners, allowing them to improve the performance of the operations.

Our research process is structured into six main parts; thus, after the introduction, further sections of the paper are organized as follows. An overview of drone realization for LTE mobile network parameter measurements is described in Section 2. Mobile network parameter impact on the controlled drone logistics is described in Section 3. Section 4 discusses the experimental setup development for our research, while Section 5 focuses on the evaluation and analysis of the measured results. Finally, the results are concluded in Section 6.

## 3. Analysis of the Mobile Network Performance Parameters for UAV Application

**Figure 1:**
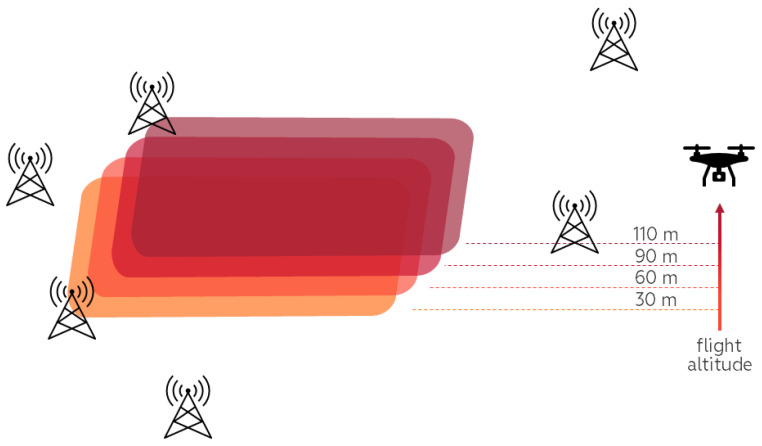
Potential drone flight trajectory and interfering antenna placement.

The mobile network architecture is usually developed to provide a stable connection for multimedia traffic transfer for a higher number of users over the longest possible distance without mobile cell interference; this task is always a balance between the requirements. To avoid interference and increase network performance, mobile operators are applying different approaches such as considering environmental properties, the relief, potential number of users, limitations to cell capacity, antennas’ possible frequency ranges and radiated power, and antennas’ spatial distribution including altitudes. Common mobile network user equipment includes mobile phones and different IoT devices, such as sensors, actuators, and other smart environment elements which are spatially distributed near ground level, while antennas are mostly placed at 15–30 m (city) and 30–100 m (rural area) altitudes and are directed at ground level, to avoid different cells interfering with each other at ground level, Figure 1.

Mobile network application for drone flight control and management has other requirements for the mobile network infrastructure, such as stable connections for multimedia traffic transfer at different altitudes and low cell interference not only around the ground but also at higher altitudes, which contradicts the requirements for the usual mobile network end users. The mobile cells’ mutual interference increases with the altitude increase, as, for example, was shown in [43,44]. Therefore, there is a challenge in the simultaneous usage of mobile networks for on-the-ground users and drones. Evaluation of the existing mobile network performance suitability for mobile network applications for controlled drone flight could answer this question or provide information for next-generation mobile network design. As drone flight should be safe for all other users, persons, living things, and infrastructure objects, it requires reliable data transmission, commands, different sensor readings, and image translation.

To be able to measure and evaluate network availability in the airspace, several important KPI parameters of the mobile network should be defined and justified by the drone-managing requirements for data transmission performance.

According to 3GPP [43], ACJA [45], and industry standards, data transmission parameters such as DL/UL and latency are defined and used in network measurement data analyses for communication performance evaluation, as well as having been analyzed in previous works [28,31,37]. However, different types of transmitted data have different sensitivities to communication-link degradation and, therefore, are observed separately. It should be noted that, according to [45], downlink (DL) and uplink (UL) terms are used as their typical meanings as they are used for cellular communications, unlike, for instance, in the field of aviation. The downlinks, in essence, are the transmissions carried out by the cellular communication tower and finished by the receiver party—UE. Uplink, in contrast, is used to describe the returning data that is sent from the UAV.

UAVs may use a variety of flight command and control modes. Command and control (C2) communications refer to the two-way communication, which may include video, required to control the operation of the UAV itself. C2 messages may be communicated with the UAV controller, the UTM, or both and may or may not be periodic. UAV controller and UTM communications may happen at essentially the same time with different required QoS. Any mission-specific communication (e.g., HD video for area surveillance), if required, is additional.

To set the KPI for DL/UL and latency, we need to observe the data-type categories of the UAV’s transmitted data. According to [45], it could be divided into non-critical communication as one of the applications and critical communication as the other, while also focusing on video as well as image streaming.

UAV operating state could be included as a non-critical communication, as well as specific configuration parameters of the UAS elements. Such information and collection could then be transmitted to the overall management system to ensure an operational situation description and provide awareness of the capabilities of the fleet devices and their performance.

Some of the non-critical communications used in the field of 4G LTE-connected UAVs can be described by usage scenarios focused on in [45].

- Telemetry of the aircraft systems—day-to-day or daily measurements received from aircraft-related subsystems (battery status, speed, heading, location/GPS), which are tiny 30 kbps messages that are sent at around 1 Hz;
- Sensor commands, messages and telemetry, where sensor command and configuration messages may follow the command protocol, requiring acknowledgment messaging and retransmitting commands multiple times before failure. Examples of this data type are ground control stations (GCS) to UAV on-board sensors supporting payload functions such as cameras for query and configuration, messages from UAV with sensor-status reports, the heartbeat of on-board sensors, and identification of individual sensors and capabilities, and commands to the UAV for image capture and confirmation of image capture from the UAV;
- Parameters/configuration messages, which are carried out periodically and as needed and consist of small data messages on GCS requesting parameter reads from UAV, GCS issues of new parameter commands, UAV broadcast parameters;
- Weather-related information, access to the airspace field, and operational volumes are some of the scenarios in non-critical communications regarding UTM messaging.

Operational control and specific command transmission to the UAV may be part of the critical communications. Such a format of data is exchanged with relatively small data rates and the specific rate used varies depending upon the system, its parameters, and other internal or external factors.

UAVs operating within the 4G LTE may be categorized for critical communications in some solutions [45] (as well as others).

- Telemetry, commands for certain missions and vehicles, and also messaging-based applications where GCS to UAV commands might consist of information regarding changes in the initial flight plans, changes in navigation settings and movement of the device, some speed related changes or other types of commands related to the fundamental operation; the category of messages concerning autopilot settings can be configured to ensure the delivery conditions (corresponding to the acknowledgments). If such a factor is not present, retransmission of the previous command communication can be executed until a new acknowledgment is approved. Such a procedure can be used to compensate for a lossy connection link.
- Heartbeat—a broadcast message received from the UAV to approve that the aircraft has stated and approved connectivity. Beyond visual-line-of-sight (BVLOS) that is LTE-connected can require a heartbeat confirmation to understand whether the UAV has the necessary connection prior to the BVLOS flight stage.
- UTM messaging, which in critical data type cases might incorporate remote ID, geofencing, and flight authorization.

It is important to note that data that is not considered a critical communication may become critical based on the UAV design. For example, video streaming may become critical if it is used for detecting and avoiding air traffic or other obstacles and, therefore, may become classified as a UTM function.

The traffic requirements for UAV applications defined by [45] for all observed data-type categories are presented in Table 2. The most significant difference between the critical and non-critical communication requirements is reliability of 10^−5^ referencing ARP4761, Guidelines and Methods for Conducting the Safety Assessment Process on Civil Airborne Systems and Equipment is an Aerospace Recommended Practice from SAE International. ARP4761 is needed to understand the evaluation framework of the critical safety data correlated to the aircraft. The FAA regulation-based framework discusses reliability requirements for data as well as safety-critical functionalities.

According to the Low-Altitude Connected Drone Flight Safety Test Report (CAAC Information Centre/Flight Standard Department/Aircraft Airworthiness Certification Department, 2018), the requirements on communication link UL/DL specifications for drone flight safety link description rate are characterized in Table 3.

Nevertheless, to maintain the relatively high credibility of aerial connections, 3GPP (the mobile broadband standard) states C2 requirements of packet delivery conditions, meaning that the packets ought to be delivered to the receiver within the set delay budget (50 ms and 99.9% reliability) in all of such scenarios. As for the C2 link and its throughput, 3GPP references 100 kbps, is thus evaluated as relatively low compared to other types of applications within the cellular field. Additionally, drones and their operational systems can also have other types of active applications that utilize or require radio communication links. For instance, live-stream videos that provide relatively high-quality images can also require high throughput connection links while ensuring the operation of the drone [47].

While the KPIs of DL/UL and latency go from requirement to reliable data transfer, these parameters for the network are secondary. These parameters depend on the overall network performance characterized by strength, quality, and interference parameters: RSRP—network strength, RSRQ—cell quality, and SINR—interference.

*RSRQ* (reference signal received quality) is a parameter that describes the ratio between *RSRP* (reference signal received power) and *RSSI* (received signal strength indicator) and is an overall indicator of the quality of signal received by the receiver. It is calculated with the following equation.

(1) $$RSRQ=\frac{N\times RSRP}{RSSI}$$

In this equation, *N* represents the number of resource blocks. *RSRP* is the power of the connection between the node on the BTS and UE.

*SINR* is the ratio between signal power and the power of noise and interference. Just like RSRQ, it is a measurement of signal quality, but unlike *RSRQ*, which only considers the strength of the signal, it also shows the strength of noise. It is calculated as follows.

(2) $$SINR=\frac{P_{signal}}{P_{noise}+P_{interference}}$$

where *P_signal_* is the signal power and *P_noise_* and *P_interference_* are the power of noise and interference, respectively.

Better overall network parameters, i.e., higher RSRP and RSRQ and lower SINR, mean the link between the UE and base station is clearer, and there is a better ability of the system to analyze the transmitted signal, so fewer errors and higher data rates in DL/UL. In turn, higher data rates provide lower ping, as the system can work more effectively, and data packets are less likely to bottleneck.

The complete table of KPIs agreed upon by LMT and RTU is visible in Table 4.

## 4. Development of the Setup for Experimental Measurements

**Figure 2:**
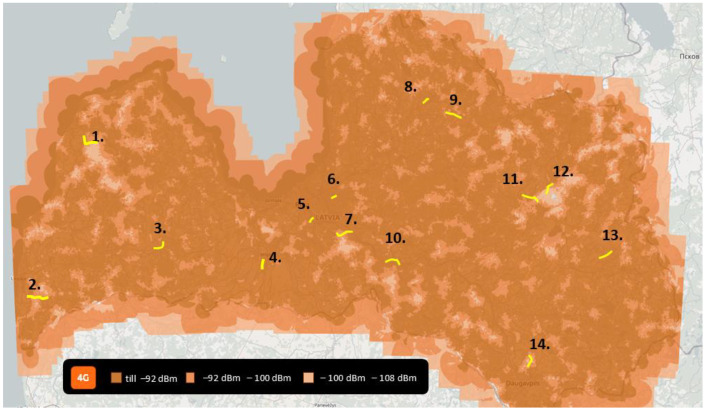
Map of measurement locations in Latvia.

In this research, 14 locations were chosen that represent various network configurations (urban/rural, dense/sparsely populated areas, wood-covered areas, and areas with good/poor network coverage on the ground). Yellow paths (in Figure 2) are locations where the flights took place covering multiple territories in Latvia, see Figure 2. In every location, the flight took place at altitudes (above take-off height) of 40 m, 60 m, 90 m, and 110 m. Each of the 14 locations had 8 flights (four different heights and two frequency modes), totaling 112 flights. The overall flight distance was approximately 700 km. The mobile network operates in different frequencies that have different propagation characteristics. As it is not feasible to test all frequencies, it was decided to focus on gathering two different flight modes, which were executed in all locations.

- Automatic mode: where the measuring device switches to the most appropriate frequency decided by mobile network algorithms.
- Band 20 (800 MHz): this is the frequency band with the best areal coverage and is very widely used.

This scientific article descriptively includes detailed information based on the flights made and measurements recorded and analyzed within route No. 1 (see Figure 2).

**Figure 3:**
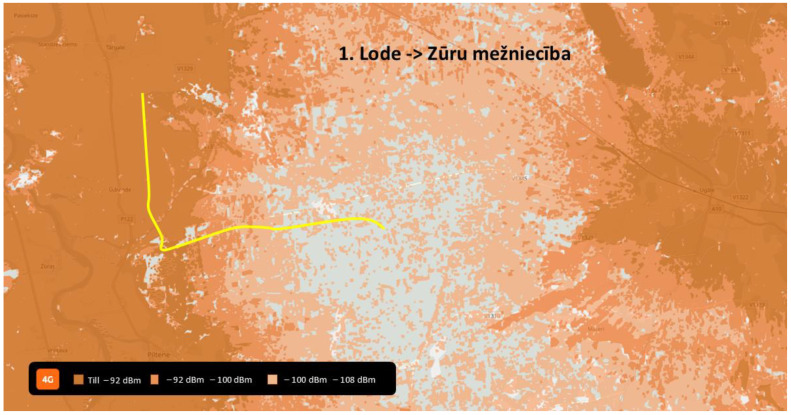
The drone’s flight path (thick yellow line).

To verify the proposed KPIs (see Table 4) of the network and satisfy the safety criteria for autonomic drone flights in the future, the testing of the network parameters was conducted at several altitudes on a specified trajectory near the towns of Lode and Piltene (see Figure 3, thick yellow line). Lode and Piltene are located northwest of the map (number 1 in Figure 2).

**Figure 4:**
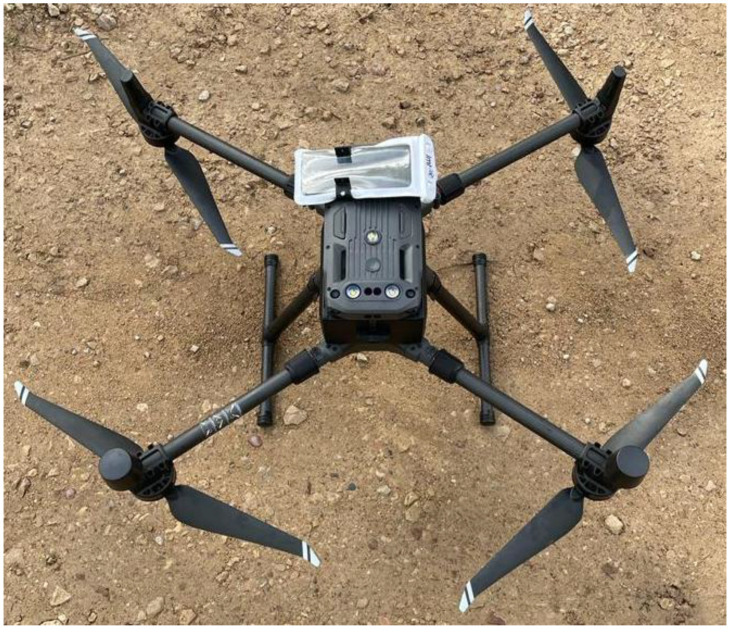
DJI M30 and OnePlus NORD BE2029 mobile phone are prepared for measurements and ready to take off.

The altitudes chosen were 40, 60, 90, and 110 m. Measurements were conducted using a OnePlus NORD BE2029 mobile phone with its original integrated antenna with the capability of operating on 2G, 3G, 4G, and 5G bands. The phone was attached to a DJI M30 drone to ensure the measurements at the set altitudes were possible. The dedicated software used was the Echo One mobile network analyzer made by Enhancell (Oulu, Finland) [48]. The image of the used setup is seen below (see Figure 4). The phone was in a waterproof case during the measurement. This was done for security reasons, e.g., deterioration of the weather would not damage the phone.

Flights were operated by an observer and pilot with supporting technical staff on-premises that prepared the flight plans and monitored the data logging process. Coordinates of the flight were from 57°16′16.3″ N 21°42′05.3″ E to 57°16′51.9″ N 21°48′37.6″ E (see in Figure 3, thick yellow line). The flight distance measured was approximately 6.7 km, which the drone completed with an average speed of 15 m/s. The weather was cloudy without rain and 17 °C.

When measurements were conducted, the mobile device had an automatic mode enabled, which ensured that the device switched between 2G, 3G, and 4G, depending on signal parameters. Based on data gathered during flights, a more accurate relation between signal KPIs (RSRP, RSRQ, and SINR) and network KPIs (latency, downlink, and uplink speed) could be determined. The UAV’s control and data transfer need to set the criteria for network KPIs and from them, the related signal KPIs can be determined.

## 5. Evaluation and Analysis of Measured Results

When focusing on the flights carried out in location “Lode”, it is possible to reach conclusions about the conjunction between certain parameters. Firstly, the conjunction between RSRQ and the other parameters was analyzed. RSRQ is one of the key metrics when it comes to assessing performance quality in mobile networks. The KPI before the measurement for RSRQ was set at −16 dB. If, during the measurement period, the value of RSRQ was consistently below this threshold, then it would be concluded that the area measured had poor signal. To test whether this threshold was adequate, the data where the RSRQ values were fluctuating around this threshold value was summarized into three categories. Data where RSRQ were between −14 and −16 dB, between −16 and −18 dB, and between −18 and −20 dB. This was carried out to see how other metrics performed if signal quality were to drop below the KPI. The other metrics were compared against their own set thresholds to see how they performed when RSRQ was recorded before the described ranges. The thresholds for other metrics were as mentioned before. RSRP: −100 dBm; SINR: −5 dB; downlink: 4096 kbps; uplink: 4096 kbps; ping: 50 ms. In Table 5, these measurements are shown for the test site Lode at measurement heights of 40, 60, 90, and 110 m. Percentages of values above the threshold are market in range: 95–100% (dark green); 94–85% (green); 84–75% (beige); <74% (pinkish).

**Figure 5:**
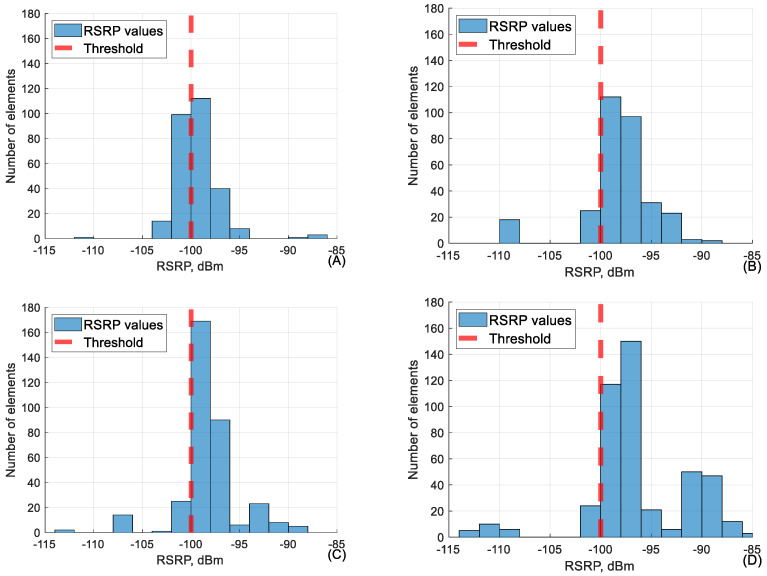
The conjunction between RSRQ and RSRP at RSRQ range (−16 to −18 dB) at (**A**) 40 m, (**B**) 60 m, (**C**) 90 m and (**D**) 110 m heights.

Table 5 shows that when looking at network parameters, the difference between the percentage of values above the RSRQ threshold, between the ranges of −14 to −16 dB and −16 to −18 dB is relatively small, meaning that even at a lower quality connection, it is possible to provide consistent network speeds and ping. It is worth mentioning that in some of the flights, it would seem that lower RSRQ yielded even better network parameters, for example, as was visible with ping at 40 m height. This is because RSRQ rarely fell into the range of −18 to −20 dB, as the handover of UE between base stations occurred shortly after RSRQ dropped and so this range had a relatively small dataset. The conjunction between RSRQ and RSRP in the range of −16 to −18 dB is shown in Figure 5.

**Figure 6:**
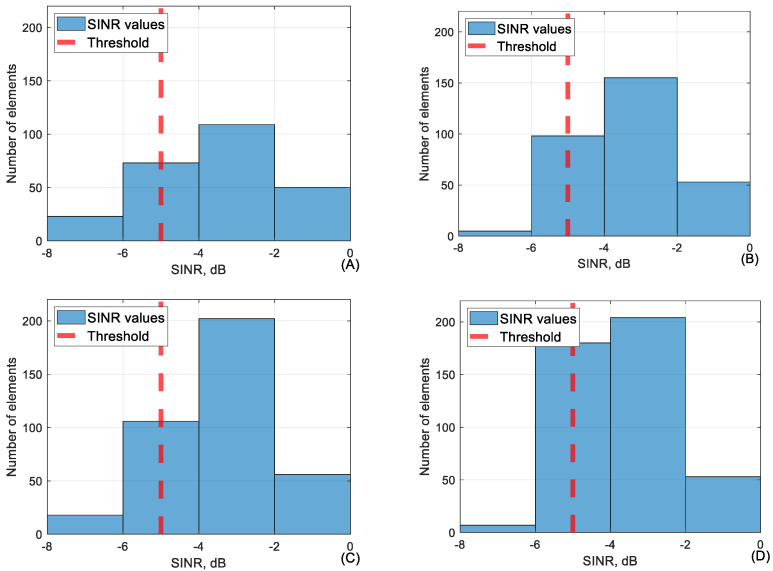
The conjunction between RSRQ and SINR at RSRQ range (−16 to −18 dB) at (**A**) 40 m, (**B**) 60 m, (**C**) 90 m, and (**D**) 110 m height.

As shown in Figure 5 and also Table 5 above, it shows that even when the value of RSRQ is below the threshold, the RSRP value is adequate, meaning above −100 dBm. The only exception is the flight at 40 m, but that is due to tree interference on signal power at lower altitudes. The following Figure 6 shows the conjunction between RSRQ and SINR in the RSRQ range of −16 to −18 dB.

**Figure 7:**
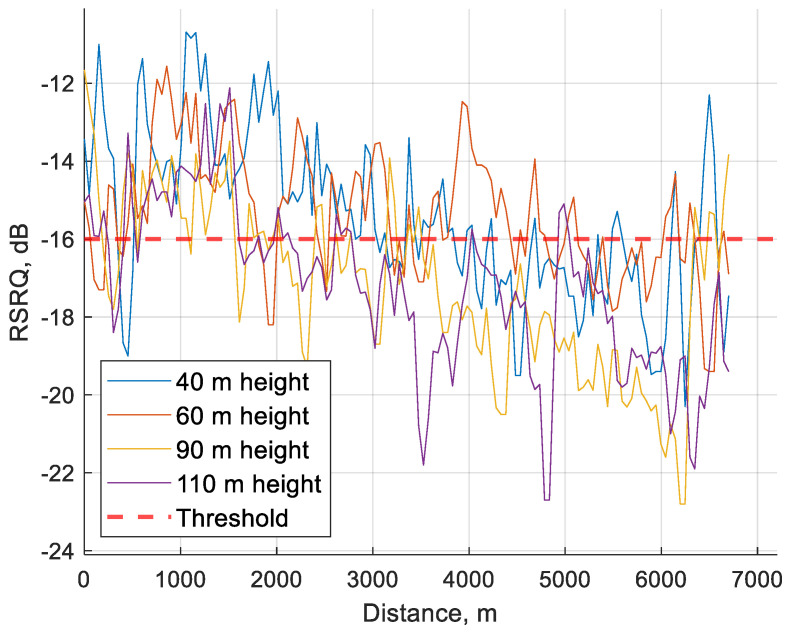
RSRQ average value over 50 m distance during the whole flights at 40, 60, 90 and 110 m heights.

Figure 6 shows that SINR values suffer slightly when RSRQ is at a lower range but in Table 5, it was visible that downlink, uplink, and ping values stayed stable regardless. The following Figure 7 shows the average value of RSRQ for every 50 m of flight.

As shown in Figure 7, during the flight, the RSRQ values fluctuated significantly and were often below the set KPI of −16 dB, but even with RSRQ falling below the set threshold, other parameters did not suffer high losses.

### 5.1. RSRP

RSRP is the power of the connection between the node on the BTS and UE. It also is essential in assessing the performance of the network but is already visible in the RSRQ equation, so RSRQ is a more inclusive parameter when it comes to network performance. Just like for RSRQ, data analysis was conducted for RSRP around its threshold value of −100 dBm. Three categories were selected with RSRP values between −95 and −100 dBm, −100 and −105 dBm, and −105 and −110 dBm. The results are shown below in Table 6. Percentages of values above the threshold are market in range: 95–100% (dark green); 94–85% (green); 84–75% (beige); <74% (pinkish).

**Figure 8:**
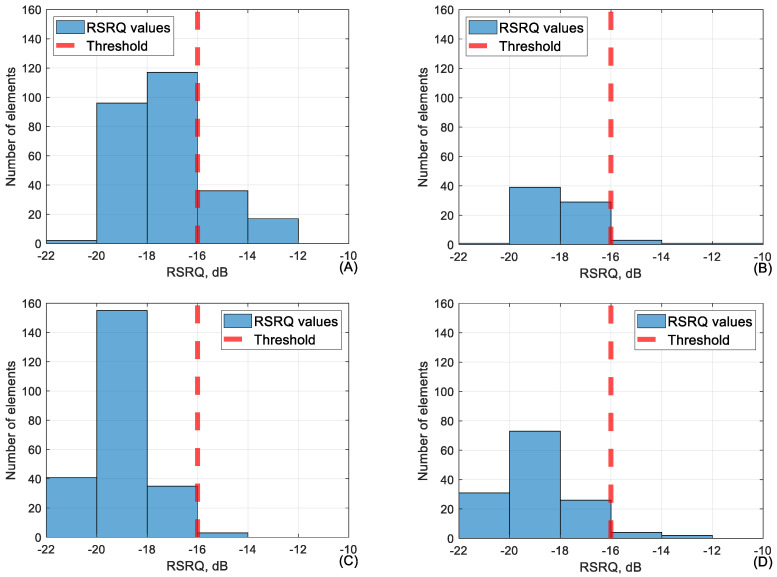
The conjunction between RSRP and RSRQ at RSRP range (−100 to −105 dBm) at (**A**) 40 m, (**B**) 60 m, (**C**) 90 m and (**D**) 110 m height.

As visible in Table 6, the network parameter values in the category of −95 to −100 dBm performed well but lower than that, the other parameters start dropping in value. It is very important to mention that during the flight, RSRP rarely falls below −100 dBm as handover procedures between base stations usually occur before that. This means that the dataset of RSRP values below −100 dBm is very small. This is the main reason why, in Table 6, the values of RSRQ and SINR adjacent to RSRP of −100 to −105 dBm seem relatively low. The conjunction between RSRP and RSRQ in the range of −100 to −105 dB is shown in Figure 8.

**Figure 9:**
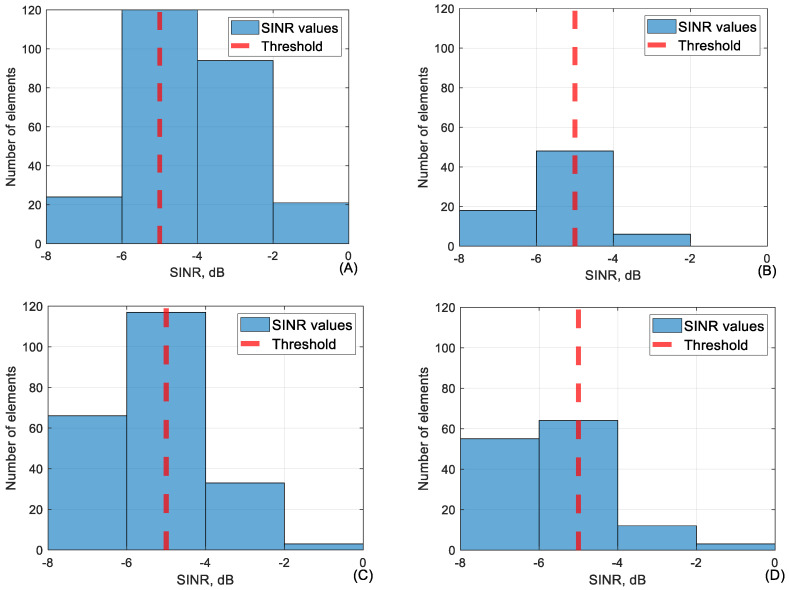
The conjunction between RSRP and SINR at RSRP range (−100 to −105 dB) at (**A**) 40 m, (**B**) 60 m, (**C**) 90 m, and (**D**) 110 m height.

As shown in the graphs (Figure 8), the measured RSRQ values are overwhelmingly below KPI at lower RSRP falling below 10% at all heights besides 40 m. This is due to the size of the dataset and the fact that signal power is one of the main variables both in RSRQ and SINR calculation. Figure 9, below, shows the conjunction between RSRP and SINR in the RSRP range of −100 to −105 dB.

Figure 9 shows that SINR values drop substantially below KPI when measuring at lower RSRP ranges. This is also shown in Table 6 above. As mentioned before, this is because of the key role of signal power in determining SINR.

**Figure 10:**
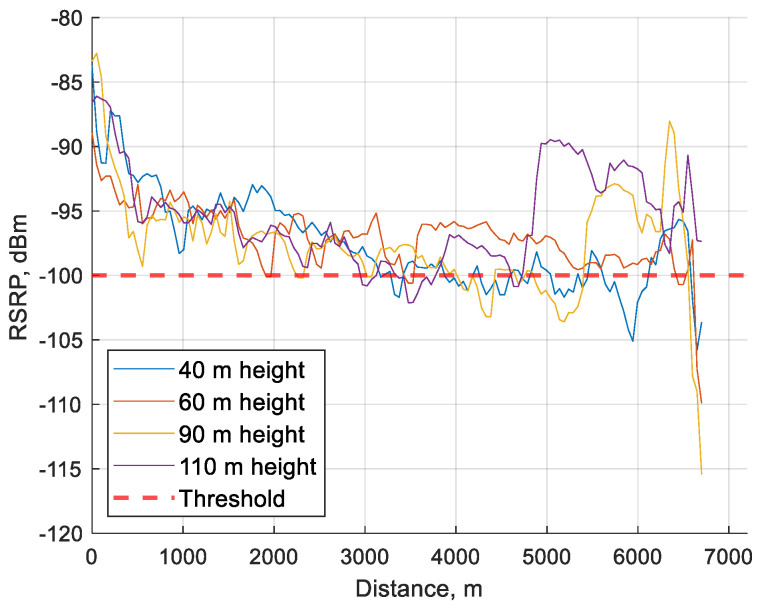
RSRP average value over 50 m distance during the whole flights at 40, 60, 90 and 110 m height.

In Figure 10, it is visible that RSRP rarely falls below the threshold, and so there is limited data in this value range, resulting in the poor RSRQ and SINR values visible in Table 6.

### 5.2. SINR

SINR is the ratio between signal power and the power of noise and interference. Just like RSRQ, it is a measurement of signal quality, but unlike RSRQ, which only considers the strength of the signal, it also shows the strength of noise. The threshold of SINR was set to −5 dB and anything below would be considered as poor performance. In the assessment of SINR, the categories were set at −2.5 to −5 dB, −5 to −7.5 dB, and −7.5 to −10 dB. This proved to be a challenge as in a regular network configuration the value of SINR will rarely drop below −5 dB as it is very likely that the UE will have connected to a different base station before this drop in SINR occurs. The results are visible below in Table 7. Percentages of values above the threshold are market in range: 95–100% (dark green); 94–85% (green); 84–75% (beige); <74% (pinkish).

**Figure 11:**
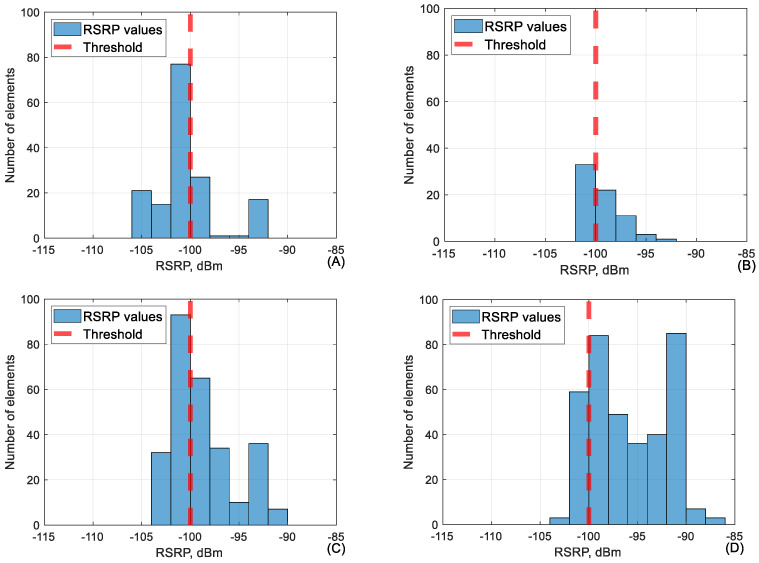
The conjunction between SINR and RSRP in SINR range (−5 to −7.5 dB) at (**A**) 40 m, (**B**) 60 m, (**C**) 90 m, and (**D**) 110 m height.

As visible, network parameters when SINR is below −5 dB can be unpredictable. For this reason, it seems that a threshold of −5 dB is sufficient to provide a reliable network. Conjunction between SINR and RSRP in the range of −5 to −7.5 dB is shown in Figure 11.

**Figure 12:**
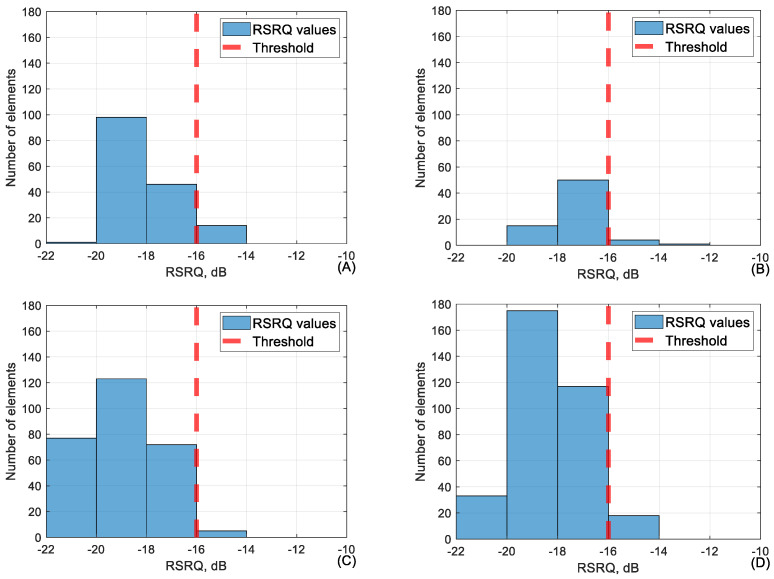
The conjunction between SINR and RSRQ in SINR range (−5 to −7.5 dB) at (**A**) 40 m, (**B**) 60 m, (**C**) 90 m, and (**D**) 110 m height.

As visible in Figure 11, RSRP is mostly below the threshold at lower SINR except for 110 m height. SINR in conjunction with RSRQ is visible in Figure 12.

**Figure 13:**
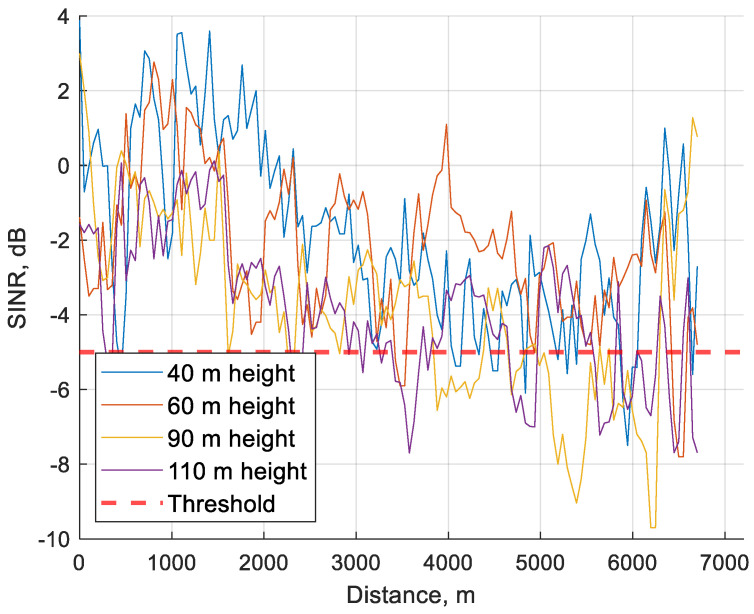
SINR average value over 50 m distance during the whole flight at 40, 60, 90 and 110 m height.

Figure 12 clearly shows that conjuring RSRQ values to lower SINR values are below the threshold and even below −18 dB as previously assessed to be an acceptable RSRQ value. In Figure 13 the average value of every 50 m of SINR is visible. As shown, the SINR value will rarely fall below −5 dB so the dataset available is limited.

To summarize, it is clear that when looking at other connection parameters, when RSRQ values are below −16 dB and in the region of −16 to −18 dB, the parameters such as RSRP, SINR, downlink, uplink, and ping meet their set KPIs with percentages similar to when RSRP is recorded at −14 to −16 dB. This means that if a connection has an RSRQ value above −18 dB, it can be considered stable. However, when RSRP and SINR values drop below their set KPI values of −100 dBm and −5 dBm, these connections become unstable and unreliable as shown in the tables above. This means that their KPI values are sufficient to provide a stable connection.

### 5.3. Recommendation

**Figure 14:**
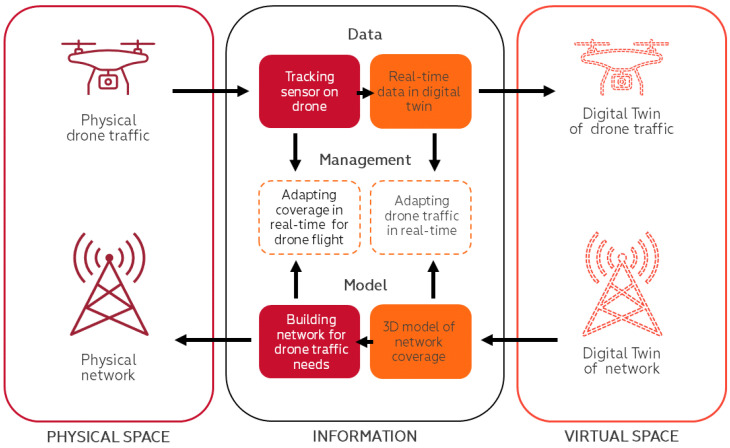
DT model of mobile network coverage and UAV flight.

For safer and more efficient UAV traffic management and applications through cellular connectivity, real-time digital twinning (DT) of mobile network coverage and performance is seen as a revolutionary technology that will undoubtedly shape the next generation of aircraft and spacecraft [49]. Therefore, mobile network measurements described in this research were also used to test the precision and usability of the DT platform for automated analytics of cellular connectivity in the airspace with flight path analysis and data for BVLOS drone operations—“AirborneRF” [50]. A model of mobile network coverage and UAV flight DT was developed to elaborate on the future research directions for the management of cellular network-enabled UAV-traffic management, see Figure 14. The set of KPI values developed could be used in the DT of mobile network coverage to plan and manage UAV traffic or adapt the network infrastructure to ensure continuous connectivity for UAV flights. According to defined KPIs, different altitudes or routes could be proposed for UAV flights. Those KPIs in DT could also serve as reference points for the deployment and upgrade of mobile network infrastructure.

DT, as a concept, was introduced in the year 2002 by Michael Grieves as a digitally connected virtual replica of the physical asset [51]. Besides various DT applications in different industries, International Telecommunication Union has also published recommendations on the requirements and architecture of a digital twin network (DTN) for analyzing, diagnosing, emulating, and controlling the physical network [52].

The proposed DT model digitally mirrors physical mobile network coverage and physical UAV, as well as UAV flight routes in real-time. This model could be used for UAV traffic management, adapting drone traffic in real-time based on the availability of network coverage and capacity. The DT model could also be used for planning or even the allocation of mobile network infrastructure in real-time depending on UAV connectivity needs.

## 6. Conclusions

As drone realization and logistics are becoming more topical with every year, in this research, an experimental study on LTE mobile network parameters for controlled drone flights was carried out. Recent and topical research trends in this topic were evaluated and therefore KPIs, such as RSRP, RSRQ, SINR, downlink, uplink, and ping, and their values were set to distinguish acceptable levels of measured parameters during experimental measurements in various configurations. The experimental setup of the DJI M30 drone and OnePlus NORD BE2029 was used at altitudes of 40, 60, 90, and 110 m.

When investigating the RSRQ parameter, it was found that in the range below the set RSRQ threshold of −16 dB (−16 to −18 dB) consistent network speeds and ping were still possible. However, RSRQ was rarely observed in the range of −18 to −20 dB and when it did, inconsistencies in other parameters appeared. Meanwhile, RSRP values still mainly reached above the set threshold of −100 dBm, even when the RSRQ was below the set KPI threshold. However, SINR values were affected negatively when RSRQ was below the set KPI but when in the RSRQ range of −16 to −18 dBm, this drop was only by about 10 percent and these effects would not result in network parameters dropping below KPI. In our analysis of RSRP and SINR, it was rarely observed that these parameters would drop below their respective thresholds of −100 dBm and −5 dBm. When these drops below threshold values were visible, inconsistencies in other parameters would emerge.

Our set KPIs, which are based on the evaluated previous research, 3GPP recommendations, RTU and LMT from previous experience, experimental study, and data gathered and shown within this article, can be further used in the business and research industries as they proved to be realistic and optimal; if the measured values are recorded outside the reference KPI zone, connections also become unstable and unreliable as shown in the data. Vast data gathered, analyzed, and shown in this research can also be used for better preparation of drone logistics and monitoring solutions.

## Figures and Tables

**Table 1:** Recent research regarding drone realization for LTE mobile network parameter measurements.

Ref.	Year	Drone/Measurement Device	Geographical Location	Environment	Application Scenario	Altitude (Meters)	Measurement
[33]	2018	Crane with multi-antenna receiver setup.	Aalborg, Denmark	City, suburbs, rural area	Multi-antenna receiver techniques for UAV communications.	0, 5, 10, 15, 20, 25, 30, 35, 40	RSP, RSRP, RSRQ, SINR
[31]	2017	Rohde & Schwarz mobile network scanner mounted underneath a commercial hexacopter drone.	Denmark	Rural area	Analysis of the radio interference for UAV connectivity over LTE.	15, 30, 60, 120	RSRP; DL SIR_0_ and DIR; DL time traces; ECCR; UL RSP; DL and UL IRC 3 dB SIR gain
[34]	2017	Portable radio network scanner (R&S TSMA0), attached underneath a commercial UAV.	Denmark	Rural area	Detection of airborne UEs based on LTE radio measurements.	1.5, 15, 30, 60, 120	RSSI, ΔRSRP, RSRP, RSRQ
[35]	2018	Quad-rotor drone with an open-source autopilot platform ArduPilo and 4G-only mobile phone for measuring latitudes, longitudes, heights, and the RSRP of 4G network.	Suzhou, China	Urban environment	4G network for air–ground data transmission: a drone-based experiment.	0–500	RSRP, packet loss rate,
[28]	2019	Drones, smart phones and Keysight Nemo Outdoor, Rohde & Schwarz QualiPoc.	Jorvas, Finland	Rural environment	Public LTE network measurements with drones in rural environments.	50, 100	UL PRBs, UL MCS class, UL data rate, UL TX power.
[36]	2019	Ultra-micro drone with Novatel 4G modem (model # USB Universal Serial Bus 720L) interfaces with the companion computer.	Irvine, USA	No information provided	Drone mapping of 4G cellular network coverage is presented using an ultra-micro drone.	30, 60, 90, 120	RSSI
[37]	2021	Commercial drone (DJI Matrice 200) and TEMS pocket application version 22.1.2 installed on the smartphone.	Flagstaff, Arizona, USA	Urban area with the highest building height of 15 m	Commercial LTE network for UAV communication in rural areas.	40, 80, 120	RSRP, RSRQ, RSSI, SINR, DL throughput, and UL throughput
[38]	2023	To investigate the signal strength of 5G, LTE, LTE-M, and NB-IoT a radio frequency (RF) measurement device TSMA6B and Skyhawk.	Odense, Denmark	No information provided	Investigation of 5G, LTE, LTE-M, and NB-IoT coverage for drone communication above 450 Feet.	137.16 m (450 feet)	RSRP, SINR
[39]	2022	Developed a cellular-connected UAV system, in which a drone is controlled by a smartphone via cellular networks.	China	Suburban environment and urban environment	Delay measurements of drones connecting to commercial LTE and 5G networks in real-world scenarios.	10, 30, 50, 80	SINR, delay
[40]	2023	SrsRAN open-source SDR software. A downward RX antenna is installed at the bottom of the UAV.	Raleigh NC, USA	Rural area	Impact of 3D antenna radiation pattern in UAV air-to-ground path loss modeling and RSRP-based localization in rural area.	30, 50, 70, 90, 110	RSRP
[41]	2023	Custom-made UAS with a DroneID5G integrated into it	Odense, Denmark	No information provided	Investigating the applicability of LTE-M for network identification of unmanned aerial systems in U-space.	20, 40, 60, 80, 100	RSSI, RSRP, RSRQ
[42]	2022	Developed multirotor drone with a smartphone and drive-test application	Selangor, Malaysia	Suburban environment	reliable aerial mobile communications with RSRP and RSRQ prediction models for the internet of drones: a machine learning approach.	65, 85, 105, 125	RSRP, RSRQ,
This research	2024	Commercial DJI M30 drone and OnePlus NORD BE2029 mobile phone with Enhancell Echo One mobile network analyzer software	Lode, Latvia	Rural area	LTE mobile network performance parameters measurements and KPI for controlled drone flights.	40,60, 90, 110	RSRQ, RSRP, SINR, Ping, DL, and UL data rates

List of used abbreviations: received signal power (RSP); interference rejection combining (IRC); signal-to-interference ratio (SIR); dominant interference ratio (DIR); physical resource blocks (PRBs); modulation and coding scheme (MCS); transmission (TX); receiver (RX), software-defined radio (SDR), estimated cell-change rates (ECCR).

**Table 2:** Traffic requirements for UAV applications [45].

Parameter	Direction	Protocol	Bandwidth (kbps)	Round-Trip Time RTT (ms)	Reliability (PLER)	Priority
Non-critical communication	UL/DL	TCP	35	500	10^−3^	System design
Critical Communication	UL/DL	TCP	35	500	10^−5^	Very high
Video and image streaming	UL	UDP	4096	500	10^−3^	System design

**Table 3:** Requirements on communication link UL/DL specifications for drone flight safety link description rate [46].

Link Description	Rate [kbit/s]	Cellular Network Latency [ms]	End-to-End Latency [ms]	Reliability	Coverage Height [m]
UL status information	30–50	50–100	<1000	10^−3^	0–1000
DL management instructions	5–10	20–50	<300	10^−3^–10^−6^	0–1000

**Table 4:** List of KPIs agreed by the LMT and RTU.

Parameter	RSRP (dBm)	RSRQ (dB)	SINR (dB)	Downlink (kbps)	Uplink (kbps)	Ping (ms)
KPI values	−100	−16	−5	4096	4096	50

**Table 5:** Percentages of values above the threshold at 40, 60, 90, and 110 m. RSRQ was between −14 and −16 dB, −16 and −18 dB, −18 and −20 dB.

RSRQ Range, dB	RSRP, %				SINR, %				Downlink, %				Uplink, %				Ping, %			
	40	60	90	110	40	60	90	110	40	60	90	110	40	60	90	110	40	60	90	110
(−14; −16)	69	84	80	55	97	99	99	97	95	97	99	99	100	97	97	100	71	93	92	78
(−16; −18)	59	86	79	90	85	86	84		90	99	94	97	100	100	99	99	86	85	84	80
(−18; −20)	22	37	50	76	34	40	34	34	100	100	99	98	97	100	99	100	97	100	61	80
										95–100%			94–85%			84–75%			<74%	

**Table 6:** Percentages of values above the threshold at 40, 60, 90, 110 m. RSRP values between −95 and −100 dBm, −100 and −105 dBm, 105 and −110 dBm.

RSRP Range, dBm	RSRQ, %				SINR, %				Downlink, %				Uplink, %				Ping, %			
	40	60	90	110	40	60	90	110	40	60	90	110	40	60	90	110	40	60	90	110
−95; −100	66	65	42	33	95	95	80	73	91	97	96	90	99	99	99	99	90	84	94	89
−100; −105	20	7	1	4	65	36	29	35	100	100	95	99	98	100	99	92	82	100	100	100
−105; −110	29	63	30	97	29	100	100	100	100	100	-	100	100	-	100	-	-	100	-	-
										95–100%			94–85%			84–75%			<74%	

**Table 7:** Percentages of values above the threshold at 40, 60, 90, and 110 m. SINR the categories were set at −2.5 to −5 dB, −5 to −7.5 dB, and −7.5 to −10 dB.

SINR Range, dB	RSRP, %				RSRQ, %				Downlink, %				Uplink, %				Ping, %			
	40	60	90	110	40	60	90	110	40	60	90	110	40	60	90	110	40	60	90	110
(−2.5; −5)	67	89	82	89	39	40	23	23	98	99	95	99	100	98	97	98	83	86	77	85
(−5; −7.5)	29	53	55	83	9	7	2	5	72	100	99	80	100	100	94	100	92	92	91	68
(−7.5; −10)	7	30	59	31	7	0	0	0	-	-	100	100	100	100	100	100	-	100	22	100
										95–100%			94–85%			84–75%			<74%	

## Data Availability

The data used to support the findings of this study are available from the first correspondence author upon request.

## References

[1] Sachs G. Drones Reporting for Work. https://www.goldmansachs.com/insights/technology-driving-innovation/drones/.

[2] Park H.C., Rachmawati T.S.N., Kim S. (2022). UAV-Based High-Rise Buildings Earthwork Monitoring—A Case Study. Sustainability.

[3] Magsino E.R., Chua J.R.B., Chua L.S., De Guzman C.M., Gepaya J.V.L. A rapid screening algorithm using a quadrotor for crack detection on bridges. Proceedings of the 2016 IEEE Region 10 Conference (TENCON).

[4] Abdallah A., Ali M.Z., Mišić J., Mišić V.B. (2019). Efficient security scheme for disaster surveillance UAV communication networks. Information.

[5] Li L., Zhang R., Li Q., Zhang K., Liu Z., Ren Z. (2023). Multidimensional Spatial Monitoring of Open Pit Mine Dust Dispersion by Unmanned Aerial Vehicle. Sci. Rep..

[6] Guo M., Sun M., Pan D., Wang G., Zhou Y., Yan B., Fu Z. (2023). High-Precision Deformation Analysis of Yingxian Wooden Pagoda Based on UAV Image and Terrestrial LiDAR Point Cloud. Herit. Sci..

[7] Saleem M.R., Mayne R., Napolitano R. (2023). Analysis of Gaze Patterns during Facade Inspection to Understand Inspector Sense-Making Processes. Sci. Rep..

[8] Vrabel J., Stopka O., Palo J., Stopkova M., Droździel P., Michalsky M. (2023). Research Regarding Different Types of Headlights on Selected Passenger Vehicles When Using Sensor-Related Equipment. Sensors.

[9] Tombari Sibe R., Bekom D. (2025). Digital Forensic Investigation of an Unmanned Aerial Vehicle (UAV): A Technical Case Study of a DJI Phantom III Professional Drone. J. Cybersecur. Inf. Manag..

[10] Ameslek O., Zahir H., Latifi H., Bachaoui E.M. (2024). Combining OBIA, CNN, and UAV imagery for automated detection and mapping of individual olive trees. Smart Agric. Technol..

[11] Barrile V., Simonetti S., Citroni R., Fotia A., Bilotta G. (2022). Experimenting Agriculture 4.0 with Sensors: A Data Fusion Approach between Remote Sensing, UAVs and Self-Driving Tractors. Sensors.

[12] Ivancic W.D., Kerczewski R.J., Murawski R.W., Matheou K., Downey A.N. (2019). Flying Drones Beyond Visual Line of Sight Using 4g LTE: Issues and Concerns. Proceedings of the 2019 Integrated Communications, Navigation and Surveillance Conference (ICNS).

[13] Almakayeel N. (2024). Flying foxes optimization with reinforcement learning for vehicle detection in UAV imagery. Sci. Rep..

[14] Huang H., Savkin A.V. (2021). Optimal Deployment of Charging Stations for Aerial Surveillance by UAVs with the Assistance of Public Transportation Vehicles. Sensors.

[15] Ezaki T., Fujitsuka K., Imura N., Nishinari K. (2024). Drone-based vertical delivery system for high-rise buildings: Multiple drones vs. a single elevator. Commun. Transp. Res..

[16] Zhai D., Wang C., Cao H., Garg S., Hassan M.M., AlQahtani S.A. (2022). Deep Neural Network Based UAV Deployment and Dynamic Power Control for 6G-Envisioned Intelligent Warehouse Logistics System. Future Gener. Comput. Syst..

[17] Huang H., Savkin A.V., Huang C. (2020). Reliable Path Planning for Drone Delivery Using a Stochastic Time-Dependent Public Transportation Network. IEEE Trans. Intell. Transp. Syst..

[18] Liu H., Yu Y., Liu S., Wang W. (2022). A Military Object Detection Model of UAV Reconnaissance Image and Feature Visualization. Appl. Sci..

[19] Mochurad L., Alsayaydeh JA J., Yusof M.F. (2024). An autopilot-based method for unmanned aerial vehicles trajectories control and adjustment. Int. J. Electr. Comput. Eng..

[20] Arif M., Hasna M.O. (2023). Analysis of Fluctuations of Antenna Pattern in U-V2X Communications. Phys. Commun..

[21] Du P., He X., Cao H., Garg S., Kaddoum G., Hassan M.M. (2023). AI-Based Energy-Efficient Path Planning of Multiple Logistics UAVs in Intelligent Transportation Systems. Comput. Commun..

[22] Salama M.R., Srinivas S. (2022). Collaborative truck multi-drone routing and scheduling problem: Package delivery with flexible launch and recovery sites. Transp. Res. Part E Logist. Transp. Rev..

[23] Sung I., Nielsen P. (2020). Zoning a Service Area of Unmanned Aerial Vehicles for Package Delivery Services. J. Intell. Robot. Syst. Theory Appl..

[24] Ahn T., Seok J., Lee I., Han J. (2018). Reliable Flying IoT Networks for UAV Disaster Rescue Operations. Mob. Inf. Syst..

[25] Wang A., Ji X., Wu D., Bai X., Ding N., Pang J., Chen S., Chen X., Fang D. (2017). GuideLoc: UAV-Assisted Multitarget Localization System for Disaster Rescue. Mob. Inf. Syst..

[26] Mayor V., Estepa R., Estepa A., Madinabeitia G. (2019). Deploying a Reliable UAV-Aided Communication Service in Disaster Areas. Wirel. Commun. Mob. Comput..

[27] Erdelj M., Natalizio E., Chowdhury K.R., Akyildiz I.F. (2017). Help from the Sky: Leveraging UAVs for Disaster Management. IEEE Pervasive Comput..

[28] Sae J., Wiren R., Kauppi J., Maattanen H.-L., Torsner J., Valkama M. (2019). Public LTE Network Measurements with Drones in Rural Environment. Proceedings of the 2019 IEEE 89th Vehicular Technology Conference (VTC2019-Spring).

[29] Alam S.S., Chakma A., Rahman M.H., Bin Mofidul R., Alam M.M., Utama I.B.K.Y., Jang Y.M. (2023). RF-Enabled Deep-Learning-Assisted Drone Detection and Identification: An End-to-End Approach. Sensors.

[30] Militaru L.G., Popescu D., Ichim L. (2020). 4G/LTE Issues of Low Altitude UAV Flying Systems. Proceedings of the 2020 24th International Conference on System Theory, Control and Computing (ICSTCC).

[31] Kovacs I., Amorim R., Nguyen H.C., Wigard J., Mogensen P. (2017). Interference Analysis for UAV Connectivity over LTE Using Aerial Radio Measurements. Proceedings of the 2017 IEEE 86th Vehicular Technology Conference (VTC-Fall).

[32] Bor-Yaliniz I., Salem M., Senerath G., Yanikomeroglu H. (2019). Is 5G Ready for Drones: A Look into Contemporary and Prospective Wireless Networks from a Standardization Perspective. IEEE Wirel. Commun..

[33] Izydorczyk T., Bucur M., Tavares F.M.L., Berardinelli G., Mogensen P. (2018). Experimental Evaluation of Multi-Antenna Receivers for UAV Communication in Live LTE Networks. Proceedings of the 2018 IEEE Globecom Workshops (GC Wkshps).

[34] Wigard J., Amorim R., Nguyen H.C., Kovacs I.Z., Mogensen P. (2017). Method for Detection of Airborne UEs Based on LTE Radio Measurements. Proceedings of the 2017 IEEE 28th Annual International Symposium on Personal, Indoor, and Mobile Radio Communications (PIMRC).

[35] Chen L., Huang Z., Liu Z., Liu D., Huang X. (2018). 4G Network for Air-Ground Data Transmission: A Drone Based Experiment. Proceedings of the 2018 IEEE International Conference on Industrial Internet (ICII).

[36] Burke P.J. (2019). 4G Coverage Mapping with an Ultra-Micro Drone. Proceedings of the 2019 IEEE Radio and Antenna Days of the Indian Ocean (RADIO).

[37] Gharib M., Nandadapu S., Afghah F. (2021). An Exhaustive Study of Using Commercial LTE Network for UAV Communication in Rural Areas. Proceedings of the 2021 IEEE International Conference on Communications Workshops (ICC Workshops).

[38] Singh R., Jepsen J.H., Ballal K.D., Nwabuona S., Berger M., Dittmann L. (2023). An Investigation of 5G, LTE, LTE-M and NB-IoT Coverage for Drone Communication above 450 Feet. Proceedings of the 2023 IEEE 24th International Symposium on a World of Wireless, Mobile and Multimedia Networks (WoWMoM).

[39] Luo J., Zhao P., Zheng F.-C., Li L. (2022). Delay Evaluation for Cellular-Connected Drones: Experiments and Analysis. Proceedings of the 2022 IEEE 96th Vehicular Technology Conference (VTC2022-Fall).

[40] Maeng S.J., Kwon H., Ozdemir O., Güvenç İ. (2023). Impact of 3-D Antenna Radiation Pattern in UAV Air-to-Ground Path Loss Modeling and RSRP-Based Localization in Rural Area. IEEE Open J. Antennas Propag..

[41] Jepsen J.H., Mader A.R., Andreasen T.D., Singh R., Jensen K. (2023). Investigating the Applicability of LTE-M for Network Identification of Unmanned Aerial Systems in U-Space. Proceedings of the 2023 International Conference on Unmanned Aircraft Systems (ICUAS).

[42] Behjati M., Zulkifley M.A., Alobaidy H.A.H., Nordin R., Abdullah N.F. (2022). Reliable Aerial Mobile Communications with RSRP & RSRQ Prediction Models for the Internet of Drones: A Machine Learning Approach. Sensors.

[43] 3rd Generation Partnership Project (2017). Technical Specification Group Radio Access Network; Study on Enhanced LTE Support for Aerial Vehicles (3GPP TR 36.777).

[44] Lin X., Wiren R., Euler S., Sadam A., Maattanen H.-L., Muruganathan S.D., Gao S., Wang Y.-P.E., Kauppi J., Zou Z. (2018). Mobile-Network Connected Drones: Field Trials, Simulations, and Design Insights. IEEE Veh. Technol. Mag..

[45] ACJA, GSMA, GUTMA LTE Aerial Profile v1.00 (ACJA, GSMA and GUTMA Approved Version). https://www.gsma.com/solutions-and-impact/technologies/internet-of-things/wp-content/uploads/2020/11/ACJA-WT3-LTE-Aerial-Profile_v1.00-2.pdf.

[46] CAAC (2018). Low-Altitude Connected Drone Flight Safety Test Report.

[47] Wigard J., Amorim R., Kovács I.Z. (2021). Controlling Drones over Cellular Networks: LTE and 5G Are Enabling Secure and Reliable Drone Control in Parallel to High Throughput Applications [White Paper].

[48] Enhancell, Echo-One. https://enhancell.com/enhancell/products/echo-one/.

[49] Souanef T., Al-Rubaye S., Tsourdos A., Ayo S., Panagiotakopoulos D. (2023). Digital Twin Development for the Airspace of the Future. Drones.

[50] AirborneRF. https://airbornerf.com/.

[51] Grieves M. (2017). Origins of the Digital Twin: Mitigating Unpredictable, Undesirable Emergent Behavior in Complex Systems.

[52] (2022). Digital Twin Network—Requirements and Architecture.

